# Age-Friendly Telegeriatrics: Evidence and a 5M-Based Framework for Hybrid Geriatric Care in Saudi Arabia

**DOI:** 10.7759/cureus.118451

**Published:** 2026-10-23

**Authors:** Saad M Alsaad

**Affiliations:** 1 Department of Family and Community Medicine, College of Medicine, King Saud University, Riyadh, SAU

**Keywords:** ageing, caregivers, comprehensive geriatric assessment, digital health, older adults, saudi arabia, telegeriatrics, telehealth, telemedicine

## Abstract

Population ageing is increasing demand for accessible, coordinated, and person-centered care, while many older adults face barriers to conventional services related to frailty, multimorbidity, cognitive or sensory impairment, functional dependency, and travel burden. Telehealth can extend specialist expertise beyond conventional clinical settings, but geriatric care cannot be reduced to transferring a standard outpatient consultation to video. This narrative review synthesizes contemporary evidence on telegeriatrics, including virtual comprehensive geriatric assessment (CGA), cognitive and functional assessment, medication review, chronic-disease monitoring, rehabilitation, home-based care, and caregiver-supported models, with particular attention to Saudi Arabia. Evidence supports the feasibility and acceptability of several telegeriatric applications, although clinical effectiveness, cost-effectiveness, and long-term outcomes remain heterogeneous. Important limitations include digital exclusion, sensory and cognitive barriers, restricted physical examination, interoperability challenges, and the risk of shifting additional work to family caregivers. Emerging Saudi evidence demonstrates both rapid expansion of virtual-care infrastructure and growing experience with telehealth among older adults, including multidisciplinary tele-CGA through Seha Virtual Hospital, but geriatric-specific outcome evidence remains limited. Based on the narrative synthesis, an author-proposed, evidence-informed conceptual framework is presented to illustrate how the Geriatric 5Ms may inform the integration of virtual and in-person geriatric care. This framework has not undergone formal consensus development, prospective validation, or effectiveness testing. It is intended to support discussion, inform future research, and provide a starting point for prospective feasibility and implementation studies rather than serve as a clinical practice recommendation.

## Introduction and background

Population ageing is reshaping healthcare needs worldwide. Older adults account for a substantial share of healthcare utilization and commonly live with multiple chronic diseases, polypharmacy, frailty, cognitive impairment, functional limitations, and complex social and caregiving needs [1-3]. Accessible geriatric care therefore requires coordinated, multidisciplinary, and longitudinal services rather than simply more clinical encounters.

Telehealth can extend specialist expertise to older adults facing geographical, mobility-related, or transportation barriers. Potential applications include chronic disease monitoring, medication review, rehabilitation, and caregiver communication. Evidence generally supports feasibility and acceptability in selected settings, but clinical effectiveness and cost-effectiveness vary across interventions and outcomes [4-7].

Providing telehealth to an older adult, however, is not equivalent to providing conventional telemedicine to a younger population. Hearing and visual impairment, cognitive decline, limited digital literacy, reduced manual dexterity, frailty, and lack of access to suitable technology may complicate virtual encounters [5,8,9]. In dementia care, telemedicine has been used for cognitive assessment, routine care, and rehabilitation, but many studies have relied on caregivers or healthcare staff to facilitate technology use [10]. Caregiver or facilitator involvement should therefore not automatically be interpreted as failure of independent telehealth use; it may be an integral component of age-friendly virtual care.

The concept of telegeriatrics extends beyond videoconferencing with an older patient. It can be understood as the application of telehealth technologies to deliver or support geriatric care while preserving the multidimensional principles of geriatrics. The geriatric 5Ms - What Matters, Medication, Mentation, Mobility, and Multicomplexity - provide a useful organizing structure for age-friendly care. Recent professional guidance has emphasized that telehealth for older adults should account for equitable access, digital literacy, cognitive and physical impairments, and caregiver support [11]. Comprehensive geriatric assessment (CGA) offers a complementary clinical framework because it integrates medical, functional, cognitive, psychological, social, and medication-related domains into an individualized care plan [12-14].

Saudi Arabia provides a particularly relevant setting in which to examine this evolution. The Kingdom has rapidly expanded digital health and national virtual-care infrastructure, including Seha Virtual Hospital (SVH) [15,16]. Saudi research has also begun to examine telehealth specifically among older adults, identifying digital literacy, connectivity, perceived usefulness, trust, family support, and facilitating conditions as important determinants of adoption [17,18]. More recently, multidisciplinary tele-CGA has been delivered through SVH to clinically complex older adults, demonstrating that virtual geriatric care can extend beyond routine consultation to multidimensional assessment [19].

Recent systematic and scoping reviews have examined telehealth effectiveness, acceptability, costs, dementia care, remote CGA, rehabilitation, and digital inclusion in older populations [4-11,20-24]. However, these studies are commonly organized around individual technologies, conditions, or outcomes rather than around the clinical logic of geriatric care. Less attention has been given to how geriatric principles should guide selection among independent virtual care, caregiver-assisted care, professionally facilitated care, hybrid assessment, and face-to-face escalation. Saudi evidence has also expanded, but it remains fragmented across adoption studies, virtual-health service evaluations, telenursing, and emerging tele-CGA experience [16-19,25].

This narrative review asks how telehealth can be organized as geriatric care rather than simply delivered to older people. It synthesizes clinical applications, implementation barriers, and Saudi evidence and then proposes an evidence-informed 5M-based hybrid-care framework. The framework is intended for prospective evaluation and is not a validated clinical prediction or triage tool.

## Review

Review approach

This clinically oriented narrative review examined telehealth and telemedicine involving older adults, geriatric applications, and the Saudi healthcare context. PubMed/MEDLINE was the primary bibliographic source. Supplementary evidence discovery used Consensus, with cited publications checked against their underlying peer-reviewed sources; reference lists of key reviews and Saudi studies were also examined. This was a topic-guided search, not a prospectively registered or exhaustive systematic search.

The literature considered publications from January 2015 through September 2026, emphasizing studies published from 2020 onward. Search concepts included telehealth, telemedicine, virtual care, remote consultation, remote monitoring, mobile health, tele-rehabilitation, older adults, ageing, geriatrics, frailty, dementia, cognition, CGA, polypharmacy, medication review, caregivers, home care, and Saudi Arabia. Publications were considered when they addressed one or more of these clinical or implementation domains. Priority was given to relevant systematic/scoping reviews, meta-analyses, randomized or comparative studies, professional guidance, and Saudi primary studies; seminal earlier CGA sources were retained. Publications unrelated to older-adult care, geriatric principles, or the Saudi virtual-care context were outside the review scope. These were topic-guided selection considerations, not prospectively applied systematic eligibility rules.

The review was narrative rather than systematic: no pooled effect estimate was planned, and study selection was based on clinical relevance to the review questions rather than a formal PRISMA screening process. The evidence was synthesized narratively by clinical domain, considering study design, population, directness to telegeriatrics, reported outcomes, and limitations. Direct telegeriatrics studies were distinguished from broader older-adult telehealth research and from conventional geriatric care evidence used for clinical rationale. Feasibility, acceptability, and satisfaction were not interpreted as proof of clinical effectiveness; observational associations were not treated as causal effects. No formal risk-of-bias scores were assigned, which limits certainty and completeness of the synthesis. The proposed framework was developed through interpretive mapping of recurring considerations (patient goals, medication safety, cognition, function, multimorbidity, digital access, human facilitation, multidisciplinary coordination, and examination/escalation needs) to the geriatrics 5Ms and care-delivery pathways. This was not a predefined or independently validated evidence-mapping procedure. Additional methodological details and limitations are given in Appendix A. OpenAI ChatGPT (GPT-5.6 Sol; OpenAI, San Francisco, CA, USA) was used as an author-support tool for literature organization, drafting assistance, language refinement, and structural editing. All cited sources, numerical statements, interpretations, and final manuscript content were reviewed and verified by the author, who assumes full responsibility for the work.

From telehealth to telegeriatrics

Telehealth encompasses synchronous video consultations, telephone-based care, asynchronous communication, remote patient monitoring, mobile-health applications, wearable devices, tele-rehabilitation, and digitally supported home healthcare. Across ageing care, these modalities have been applied to cardiovascular and chronic disease management, mental health, cognitive impairment, frailty, rehabilitation, and other domains [4-7]. The clinical value of these technologies depends less on the platform itself than on whether they enable the core objectives of geriatric care. Table 1 summarizes the principal telehealth modalities and their potential geriatric applications. Older adults of the same chronological age can differ markedly in functional reserve, cognition, multimorbidity, social support, and capacity for self-management. A telegeriatric service therefore needs to determine not only whether an encounter can occur remotely but also how it should occur and what assistance is required. Digital independence should not be the sole criterion for access.

**Table 1:** Telehealth modalities and potential applications in geriatric care

Modality	Geriatric applications	Potential advantages	Important limitations
Video consultation [5,19,20]	CGA, chronic disease, cognition, medication review, caregiver consultation	Visual interaction, specialist access, home context	Sensory/cognitive impairment, examination limitations
Telephone [5,19,22]	Follow-up, medication review, selected cognitive tools, caregiver support	Low technological requirement	No visual examination, hearing impairment
Remote patient monitoring [4]	BP, glucose, weight, oxygen saturation, chronic-disease monitoring	Longitudinal surveillance, potential early detection	Device burden, alert fatigue, response pathway required
Tele-rehabilitation [23]	Exercise, mobility, balance, functional interventions	Home access, continuity	Safety and supervision, low-certainty effectiveness evidence
Caregiver-assisted telehealth [10,19]	Dementia, frailty, medication reconciliation, function	Enables participation of dependent patients	Privacy, autonomy, caregiver workload
Professionally facilitated telehealth [19,20]	Tele-CGA, home healthcare, complex frailty	Specialist access plus local hands-on assessment	Staffing and coordination requirements
Hybrid care [5,11,20,21]	Complex assessment and longitudinal management	Preserves access while allowing direct examination	Integrated scheduling and escalation required

This distinction is particularly important for CGA. Several components of CGA can be performed remotely, including history, medication review, cognitive screening, mood assessment, activities of daily living (ADL)/instrumental activities of daily living (IADL) assessment, frailty estimation, social assessment, and caregiver evaluation. Wu et al. described the potential for tele-CGA to improve accessibility and monitoring while emphasizing technological, data-security, and comprehensiveness challenges [20]. A systematic review of digital technologies supporting CGA similarly identified usability, connectivity, hardware access, assessment length, and professional training as implementation barriers [21].

A practical distinction can therefore be made among independent telegeriatrics, caregiver-assisted telegeriatrics, healthcare professional-facilitated telegeriatrics, and hybrid geriatric care. These models are not mutually exclusive; an older adult may move among them as clinical condition, cognition, function, or available support changes.

The 5Ms of geriatric care provide a useful clinical anchor for this approach: What Matters, Medication, Mentation, Mobility, and Multicomplexity [11]. Of these, What Matters is especially important when deciding whether virtual care is appropriate. Digital readiness determines whether telehealth is technically possible; What Matters helps determine whether it is desirable. Avoiding travel, remaining at home, involving or excluding a caregiver, preserving privacy, and preferring face-to-face interaction may all influence the appropriate mode of care even when virtual consultation is technically feasible.

Clinical applications of telegeriatrics

Virtual Comprehensive Geriatric Assessment

Virtual CGA may be one of the most clinically important applications of telegeriatrics because it addresses the multidimensional needs that distinguish geriatric medicine from disease-specific teleconsultation. It may be particularly useful for homebound patients, individuals with mobility limitations, patients living far from geriatric services, and those already receiving home healthcare.

The evidence should nevertheless be interpreted carefully. Evidence supporting conventional or home-based CGA cannot automatically be assumed to demonstrate the effectiveness of tele-CGA. Digital and telemedicine-based CGA remain heterogeneous in content, staffing, technology, and setting [20,21]. Virtual CGA is therefore best viewed as an evolving method of extending geriatric expertise rather than a universally interchangeable substitute for conventional CGA.

The SVH tele-CGA service illustrates a multidisciplinary approach in practice. The model uses sequential video-based assessment involving a consultant geriatrician, a geriatric-trained nurse, and a clinical pharmacist. It includes cognitive screening, mental-health evaluation, ADL/IADL assessment, social circumstances, medication review, and a written care plan [19]. Remote tools include telephone-adapted cognitive measures, AD8, Mini-Cog, Katz ADL, and the Clinical Frailty Scale; gait may be observed by video when feasible. Direct hands-on examination and orthostatic blood-pressure measurement remain limitations when trained personnel are not physically present [19].

Cognitive Assessment and Dementia Care

Cognitive impairment is both an important indication for telegeriatrics and a major implementation challenge. Remote access may reduce travel burden and improve access to memory or geriatric expertise, yet cognitive impairment can reduce the ability to navigate platforms, provide reliable history, or follow testing instructions. Evidence supports the feasibility of selected remote cognitive assessments, but validity depends on the specific instrument, mode of administration, sensory function, and testing environment [10,22].

Yi et al. found telemedicine applications for cognitive assessment, routine dementia care, and rehabilitation; notably, most included studies relied on staff or care partners to help participants navigate technology [10]. Hearing impairment and exclusion of people with sensory limitations were also reported. Requiring independent technological competence could therefore systematically exclude some of the older adults most likely to benefit from reduced travel and specialist access.

Caregivers can provide collateral history, clarify medication use, describe changes in cognition and behavior, and assist with functional and safety assessment. Their participation should complement rather than automatically replace the older person. Privacy, consent, autonomy, and differences between patient and caregiver perspectives remain important. Cognitive impairment should consequently prompt adaptation of the telehealth model, not automatic exclusion from it.

Frailty, Functional Status, Mobility, and Falls

Frailty and functional status influence prognosis, treatment tolerance, hospitalization risk, independence, and the intensity of healthcare interventions. ADL and IADL can be assessed remotely through structured interviews, and selected frailty measures can be applied when adequate clinical information is available. Transfers and gait may sometimes be observed by video, but camera positioning, safety, and environmental constraints affect reliability.

Falls assessment illustrates the need for hybrid care. History, medication review, cognition, environmental hazards, and selected mobility tasks may be assessed remotely, but a complete evaluation may require orthostatic measurements, neurological or musculoskeletal examination, vision assessment, and direct balance testing. Telehealth can therefore support falls assessment without replacing all in-person components. Table 2 summarizes the relative suitability of virtual care across common geriatric domains and situations in which hybrid or in-person assessment is often required.

**Table 2:** Clinical suitability of telegeriatrics across common geriatric domains

Domain	Virtual-only suitability	What telegeriatrics can reasonably provide	When hybrid/in-person assessment is often needed
CGA [20,21]	Conditional	Medical, medication, cognitive, mood, functional, social, goals and caregiver assessment	Hands-on examination, unresolved diagnostic questions, unstable or highly complex presentations
Cognition/dementia [10,22]	Conditional	Selected remote cognitive testing, collateral history, follow-up	Severe sensory/communication barriers, uncertain diagnosis, safety concerns, lack of needed facilitation
Frailty/function [19,20,23]	Conditional	Interview-based measures, ADL/IADL, selected video observation	Performance testing or examination that cannot be safely or reliably completed remotely
Falls [5,20,23]	Low-conditional	History, medication review, environmental observation, selected mobility tasks	Orthostatic measurements, neurological/cardiovascular examination, syncope, recurrent unexplained falls
Polypharmacy [19,26,27]	High	Medication reconciliation, adherence review, deprescribing discussion, pharmacist input	Suspected adverse event requiring examination, monitoring, or urgent investigation
Stable chronic disease [4,6,28]	High-conditional	Follow-up and selected remote physiological monitoring	Acute deterioration, new unexplained symptoms, examination-dependent decisions
Rehabilitation [23]	Conditional	Selected supervised exercise, education, progression monitoring	Unsafe home environment, high falls risk, need for hands-on assessment or assistance
Home healthcare [20]	High when facilitated	Specialist input supported by local nursing or allied health professionals	Clinical instability or needs beyond available home-based assessment/intervention
Caregiver support [10,19]	High	Education, collateral history, technology facilitation, burden assessment	Safeguarding, conflict, privacy/autonomy concerns requiring separate or direct assessment

Tele-rehabilitation and remotely supervised exercise may complement geriatric care. A systematic review and meta-analysis of telehealth-delivered exercise interventions for older adults with frailty, cognitive impairment, or mobility disability found signals of benefit in mobility, strength, and balance, but pooled between-group effects were not consistently significant, and certainty of evidence was low or very low [23]. These findings do not establish equivalence or superiority to in-person rehabilitation. Numerical pooled estimates should be interpreted from the original review rather than inferred from feasibility findings.

Polypharmacy and Medication Review

Polypharmacy is common in older adults and contributes to adverse drug events, interactions, treatment burden, falls, and potentially inappropriate prescribing. Medication review is particularly suitable for multidisciplinary telegeriatrics because much of the process depends on accurate medication history, indications, dose, laboratory context, adherence, and adverse effects rather than direct physical examination.

Virtual encounters can facilitate home medication reconciliation by allowing patients or caregivers to show medication containers, over-the-counter products, supplements, inhalers, or injectable devices. A systematic review of multidisciplinary interventions in community-dwelling older adults reported improvements in medication appropriateness and medication burden, while clinical endpoints such as falls, mortality, function, and utilization were less consistently improved [26]. Because these interventions were not necessarily delivered remotely, they support the clinical rationale for medication review rather than demonstrate telepharmacy effectiveness.

Within the SVH tele-CGA model, a clinical pharmacist participates in medication review, which is particularly relevant in a cohort characterized by substantial polypharmacy [19]. Digital medication-management interventions are likely to depend on usability, interoperability, workflow integration, and multidisciplinary clinical ownership rather than technology alone [27].

Multimorbidity, Chronic Disease, and Remote Monitoring

Multimorbidity is characteristic of geriatric practice and challenges disease-specific telehealth models. An older adult may simultaneously require management of diabetes, hypertension, cardiovascular disease, kidney disease, cognitive impairment, pain, functional decline, and multiple medications. Telegeriatrics should therefore coordinate disease management around overall goals, function, treatment burden, and prognosis rather than reproduce multiple isolated virtual clinics.

Remote patient monitoring can complement virtual consultation through longitudinal measurement of blood pressure, glucose, heart rate, oxygen saturation, body weight, activity, or other clinically relevant parameters. In the eCOBALTH randomized trial of older adults with multimorbidity following hospitalization, a 12-month home telesurveillance program was associated with fewer unplanned hospitalizations than usual care [28]. However, remote-monitoring programs vary substantially in technology, intensity, response pathways, and patient selection, limiting broad generalization.

Monitoring is clinically meaningful only when data are linked to an accountable response system. Services should define what is monitored, who reviews incoming information, what constitutes an alert, how rapidly clinicians respond, and when the patient should transition to in-person or emergency care.

Tele-Rehabilitation and Home Healthcare

The intersection between telegeriatrics and home healthcare is especially relevant for people with advanced frailty, disability, or difficulty attending outpatient services. Home-healthcare professionals can serve as the physical extension of the remote geriatric team by obtaining vital signs, assessing wounds, reviewing medications, evaluating the home environment, performing selected functional assessments, and facilitating communication with specialists.

A scoping review of digital home-care interventions for older adults highlighted potential benefits for access and care quality while emphasizing digital literacy, infrastructure, caregiver access, and patient involvement as implementation priorities [29]. This supports a model in which virtual specialist expertise is integrated with local hands-on care rather than treated as a replacement for it. Saudi-specific effectiveness and cost-effectiveness evidence for such integrated models remains limited and should be a research priority.

The Caregiver as Part of the Telegeriatrics Model

Conventional telehealth frameworks often conceptualize the encounter as a relationship between patient, clinician, and technology. For many frail older adults, a more realistic model is a patient-caregiver-clinician relationship supported by technology. The caregiver may occupy two distinct roles that should not be conflated: facilitator of telegeriatrics and recipient of care. As a facilitator, the caregiver may support technology use, provide collateral history, reconcile medications, describe cognition and function, and help implement the care plan. As a recipient of care, the caregiver may require education, psychological support, competence-building, respite planning, and assessment of burden.

The need for caregiver involvement becomes particularly evident in highly dependent populations. In the SVH tele-CGA cohort, substantial proportions of patients were dependent in IADLs or bedbound, making a model based exclusively on independent patient interaction unrealistic [19]. Yet reliance on caregivers has consequences. Telehealth may reduce transportation demands while shifting responsibilities to the family, including technology setup, monitoring, medication management, and communication with clinicians.

Caregiver burden should be assessed rather than assumed away. In the Saudi SVH tele-CGA cohort, 146 caregiver respondents were included in the cross-sectional analysis. Patient difficulty in usual activities was associated with greater caregiver burden (adjusted odds ratio [aOR] 4.99, 95% CI 1.56-16.03), as were pain/discomfort (aOR 2.66, 95% CI 1.08-6.55) and anxiety/depression (aOR 2.42, 95% CI 1.09-5.35) [19]. These associations do not show that telemedicine caused or reduced caregiver burden.

Telehealth can also be directed toward caregivers themselves. A recent systematic review and meta-analysis of interventions for family caregivers of older adults with multiple diseases found short-term improvements in psychological well-being, caregiving competence, and caregiver burden, although longer-term benefits were less consistent [30]. An age-friendly telegeriatrics service should therefore ask not only whether a caregiver is available but also whether the caregiver is willing and able to participate, understands the care plan, requires training, or needs support.

Potential benefits and outcomes

The most immediate potential benefit of telegeriatrics is improved access to geriatric expertise. Virtual care can reduce geographical and transportation barriers and may be particularly valuable for homebound people, patients with mobility limitations, and those who require family members to accompany them. It may also improve continuity by allowing follow-up without repeated travel.

Systematic reviews generally report favorable feasibility and satisfaction, but clinical effectiveness varies by intervention, population, comparator, and outcome [4-7]. This distinction matters. Telegeriatrics does not need to outperform every face-to-face encounter to be valuable; comparable outcomes may represent meaningful benefit when specialist care would otherwise be difficult to access. Conversely, high satisfaction alone does not establish clinical effectiveness.

Economic evaluation requires similar caution. Virtual encounters may reduce travel and potentially improve use of specialist time, but telehealth also requires infrastructure, technical support, devices, cybersecurity, integration with electronic records, training, and clinical monitoring. A recent systematic review found generally high satisfaction and potential cost savings in selected geriatric telehealth studies but emphasized heterogeneity and limited evidence, especially in the oldest age groups [24].

Future evaluations should move beyond visit counts. Relevant outcomes include access, waiting time, completion of CGA recommendations, medication appropriateness, functional trajectory, falls, emergency visits, hospitalization, institutionalization, quality of life, caregiver burden, patient and caregiver experience, equity of access, and total healthcare utilization.

Challenges and risks

Digital exclusion is among the most important concerns. Older adults are heterogeneous in experience with smartphones, applications, video platforms, and connected devices. Limited digital literacy may coexist with lower educational attainment, cognitive impairment, sensory loss, socioeconomic disadvantage, or rural residence. Services designed primarily for technologically confident older adults risk widening rather than reducing inequalities [5,8,9].

Sensory and cognitive impairments require deliberate adaptation. Hearing loss may make telephone or video consultation difficult, visual impairment may interfere with reading instructions or operating devices, and cognitive impairment may affect navigation and comprehension. Age-friendly interfaces should therefore prioritize simplicity, readable displays, accessible audio, minimal navigation steps, and appropriate caregiver or facilitator access.

Clinical limitations are equally important. Remote consultation cannot reliably reproduce palpation, auscultation, detailed neurological examination, assessment of rigidity, some gait and balance procedures, orthostatic measurements, or diagnostic testing. Poor video quality can further reduce reliability. These limitations support explicit hybrid pathways rather than an assumption that all care can be virtual.

Privacy and autonomy require particular attention when caregivers are present. Some patients may not wish to discuss mood, family conflict, abuse, financial concerns, continence, or other sensitive issues in front of relatives. Clinicians should preserve opportunities for private communication where feasible and appropriate.

Finally, fragmentation can undermine otherwise successful telehealth programs. A virtual specialist recommendation has limited value if it is invisible to primary care, cannot modify prescriptions, is disconnected from home healthcare, or lacks a mechanism for arranging investigations and face-to-face follow-up. Interoperability and clinical integration are therefore core clinical requirements of telegeriatrics, not merely information-technology considerations.

Telegeriatrics in Saudi Arabia

Saudi Arabia provides a distinctive environment for telegeriatrics because rapid expansion of national virtual-care capability is occurring alongside increasing demand for chronic-disease and older-adult services. SVH represents an important component of this infrastructure. A retrospective national service analysis documented substantial expansion in participating centers, workforce, beneficiaries, and virtual outpatient activity between 2022 and 2024 [16]. This demonstrates system capacity for virtual specialist care but should not be interpreted as evidence of geriatric effectiveness.

Saudi studies focused specifically on older adults identify implementation issues that are directly relevant to age-friendly design. In qualitative interviews with 25 adults aged 60 years or older in Riyadh, Alodhialah et al. identified digital literacy, connectivity, trust, support systems, and institutional factors as important influences on telehealth adoption [17]. In Jazan, Meraya et al. studied 466 older adults with chronic conditions and found that perceived usefulness and facilitating conditions were strongly associated with attitudes toward telehealth [18]. Alharthi further demonstrated that digital readiness is a multidimensional consideration among older adults in Saudi Arabia; persistent usability, digital literacy, accessibility, privacy, and infrastructure barriers can limit independent mHealth use, while family support may facilitate access and engagement [31]. More recent evidence from southwestern Saudi Arabia provides additional insight into these challenges. Alqahtani and Almaghaslah studied 500 adults aged ≥60 years in Aseer and found generally favorable attitudes toward mHealth, while identifying age-related differences in awareness and continuing digital-literacy and usability concerns [32]. Hawash similarly identified personal, technical, and environmental barriers to digital-device use among 400 community-dwelling older adults in Abha, with greater barriers among several vulnerable groups, including the oldest adults and those with lower education, lower income, or rural residence [33]. Aljohani's study of older adults with disability or their caregivers further suggested demand for selected telenursing services while demonstrating heterogeneity according to health and sociodemographic characteristics [25]. Together, these studies argue against treating older Saudi adults as a homogeneous group of technology users.

Saudi evidence has also begun to move from adoption toward geriatric clinical practice. The SVH tele-CGA service uses a multidisciplinary model involving a consultant geriatrician, geriatric-trained nurse, and clinical pharmacist, with assessment of cognition, mood, function, frailty, medications, social circumstances, and caregiver needs [19]. The associated cohort was clinically complex, with substantial multimorbidity, polypharmacy, functional dependency, and multiple geriatric syndromes [19]. This is important because it demonstrates the feasibility of delivering a multidimensional geriatric service to patients who are more complex than the relatively independent populations represented in many general telehealth studies.

Family involvement is another important implementation consideration. In the SVH cohort, high levels of IADL dependency and bedbound status meant that many encounters could not realistically be conceptualized as independent patient-to-clinician teleconsultations [19]. Caregiver burden was also common, and poorer patient quality-of-life domains were associated with greater caregiver burden in adjusted analyses [19]. These findings do not establish that telemedicine causes or reduces caregiver burden; rather, they show that caregiver capacity is part of the clinical context in which telegeriatrics operates.

The current Saudi evidence base nevertheless remains limited and methodologically heterogeneous. Adoption studies are primarily cross-sectional or qualitative, national virtual-hospital evaluations are not geriatric-specific, and the tele-CGA evidence is observational. There is limited Saudi evidence on comparative clinical effectiveness, cost-effectiveness, functional trajectories, falls, medication-related outcomes, rural access, implementation fidelity, or long-term caregiver outcomes. Evidence from digitally connected urban populations may also underrepresent older adults with low literacy, sensory impairment, limited connectivity, or little family support. The principal Saudi studies relevant to age-friendly telegeriatrics, together with their limitations and contribution to the proposed framework, are summarized in Table 3.

**Table 3:** Current Saudi evidence relevant to age-friendly telegeriatrics and remaining evidence gaps mHealth, mobile health; tele-CGA, telemedicine-based comprehensive geriatric assessment; SVH, Seha Virtual Hospital

Study	Design / setting	Population	Main finding	Key limitation	Relevance to framework
Alodhialah et al., 2024 [17]	Qualitative; Riyadh	25 adults aged ≥60 years	Digital literacy, connectivity, trust, support and institutional factors influence adoption	Small qualitative sample; urban setting	Digital readiness; family/support layer
Meraya et al., 2025 [18]	Cross-sectional; Jazan	466 older adults with chronic conditions	Perceived usefulness and facilitating conditions were important determinants of attitudes	Cross-sectional; attitudes rather than clinical outcomes	Readiness, usability, and facilitating conditions
Aljohani, 2025 [25]	Cross-sectional; Saudi Arabia	188 older adults with disability or caregivers	Demand for selected telenursing services varied by health and sociodemographic factors	Demand does not establish effectiveness	Need for tailored rather than uniform virtual services
Alharthi, 2025 [31]	Repeated cross-sectional, multimethod study; Saudi Arabia	2021 family survey (n=397), interviews with older adults (n=5), observations; 2024 older-adult survey (n=129); review of 21 mHealth apps	Persistent digital-literacy, usability, accessibility, privacy, and infrastructure barriers limited independent mHealth use. Family and social support facilitated access, while post-pandemic users recognized useful health functions despite continuing technical and trust concerns.	Repeated cross-sectional rather than longitudinal; self-reported data; heterogeneous data sources and age definitions; no clinical effectiveness outcomes.	Digital readiness, human support, caregiver-assisted care, accessibility, privacy, equity
Alqahtani and Almaghaslah, 2025 [32]	Cross-sectional study; Aseer Region, Saudi Arabia	500 adults aged ≥60 years	Older adults reported generally favorable attitudes toward mHealth, while awareness and use varied across sociodemographic characteristics; digital-literacy and usability issues remained relevant barriers.	Cross-sectional design, convenience sampling, and attitudes and use rather than clinical outcomes	Digital readiness, usability, age-friendly design, acceptability
Hawash, 2025 [33]	Cross-sectional study; Abha, Saudi Arabia	400 community-dwelling adults aged ≥60 years	Identified personal, technical, and environmental barriers to digital-device use, with greater barriers among several vulnerable groups, including the oldest adults and those with lower education, lower income, or rural residence.	Cross-sectional design; focuses on digital device use rather than telehealth clinical outcomes	Digital readiness, equity, accessibility, targeted support
Alabdulaali et al., 2026 [16]	Retrospective service analysis; SVH	National virtual-hospital activity	Documents rapid expansion of national virtual-health infrastructure	Not geriatric-specific, descriptive outcomes	System capacity for specialist virtual care
Khusheim et al., 2026 [19]	Cross-sectional tele-CGA; SVH	Older adults receiving tele-CGA; 146 caregiver survey respondents	Demonstrates multidisciplinary tele-CGA in a clinically complex population and substantial caregiver burden	Observational, no comparator, cannot establish tele-CGA effectiveness	CGA core, caregiver role, multidisciplinary integration

The immediate opportunity in Saudi Arabia is therefore not simply to increase the number of virtual consultations delivered to older adults. It is to connect existing virtual infrastructure with primary care, geriatrics, clinical pharmacy, home healthcare, rehabilitation, and caregiver support in a model that can vary the intensity of human assistance and move bidirectionally between virtual and face-to-face care. The proposed framework below is intended to organize that transition and to make the remaining evidence gaps explicit.

The recurring evidence concerns were mapped conceptually to framework components: patient goals to What Matters; medication safety to Medication; cognition, communication, and digital accessibility to Mentation and facilitated delivery; frailty, falls, and examination limitations to Mobility and hybrid review; and multimorbidity, social circumstances, and caregiving demands to Multicomplexity. Evidence on service fragmentation informed multidisciplinary coordination, while uncertainty and clinical deterioration informed reassessment and escalation. These connections are interpretive proposals, not tested causal relationships or validated triage rules.

Proposed 5M-based age-friendly telegeriatrics framework for Saudi Arabia

The proposed framework places the geriatric 5Ms at the clinical core of telegeriatrics and surrounds them with an implementation layer addressing digital readiness, caregiver support, professional facilitation, multidisciplinary integration, and escalation. Technology is treated as an enabling mechanism within a geriatric care pathway rather than the organizing principle of care.

Step 1: Clinical and Digital Readiness Assessment

Suitability for virtual care should be assessed across both clinical and digital dimensions. Clinical considerations include the reason for referral, stability, frailty, cognition, function, sensory impairment, communication ability, mobility, and the need for direct examination or testing. Digital considerations include device and connectivity access, ability to use the platform, technical support, and availability of a caregiver or healthcare professional. The result is not a binary eligible/ineligible decision but selection among independent virtual care, caregiver-assisted care, professionally facilitated care, or hybrid/in-person care.

Step 2: Pre-Visit Preparation

Before the encounter, services should confirm the referral question, medication list, diagnoses and investigations, caregiver details, technology readiness, and available home measurements. Selected screening information on ADL/IADL, cognition, frailty, falls, nutrition, and mood may be collected in advance. When home-healthcare or primary-care professionals facilitate the encounter, vital signs and selected physical assessments can be obtained locally.

Step 3: Virtual Assessment Organized Around the 5Ms

The virtual geriatric assessment should explicitly address What Matters, Medication, Mentation, Mobility, and Multicomplexity. What Matters anchors the encounter in the older person's goals and priorities. Medication addresses polypharmacy, appropriateness, adherence, and treatment burden. Mentation includes cognition, delirium risk, mood, and behavioral symptoms. Mobility encompasses function, frailty, gait, falls, and rehabilitation needs. Multicomplexity integrates multimorbidity, social circumstances, caregiving, prognosis, and the interaction among these domains. The depth of assessment should be individualized rather than identical for every patient.

Step 4: Multidisciplinary Integrated Management

Assessment should connect directly to action. Depending on identified needs, telegeriatrics should coordinate with primary care and family medicine, clinical pharmacy, home healthcare and nursing, rehabilitation and physiotherapy, mental-health services, social care, and relevant specialties. This creates a distributed model in which expertise can be delivered virtually while interventions occur as close as possible to the patient's home.

Step 5: Escalation and Hybrid Care

A safe model requires explicit recognition of the limits of remote care. Direct examination, diagnostic testing, acute deterioration, or uncertainty should trigger planned escalation to primary care, geriatric outpatient review, home-healthcare assessment, emergency evaluation, hospital admission, or another specialty service. The pathway should remain bidirectional: virtual care, facilitated virtual care, face-to-face care, and acute care are connected modes within one system rather than separate services.

Step 6: Longitudinal Monitoring and Reassessment

Suitability for telehealth changes over time. Follow-up may combine virtual consultation, medication review, caregiver assessment, functional reassessment, and selected remote monitoring. Outcomes should be tracked at patient and service levels, including function, quality of life, falls, medication burden, utilization, caregiver burden, waiting time, failed virtual encounters, conversion to in-person care, experience, and equity of access.

Figure 1 shows the proposed 5M-based age-friendly telegeriatrics.

**Figure 1:**
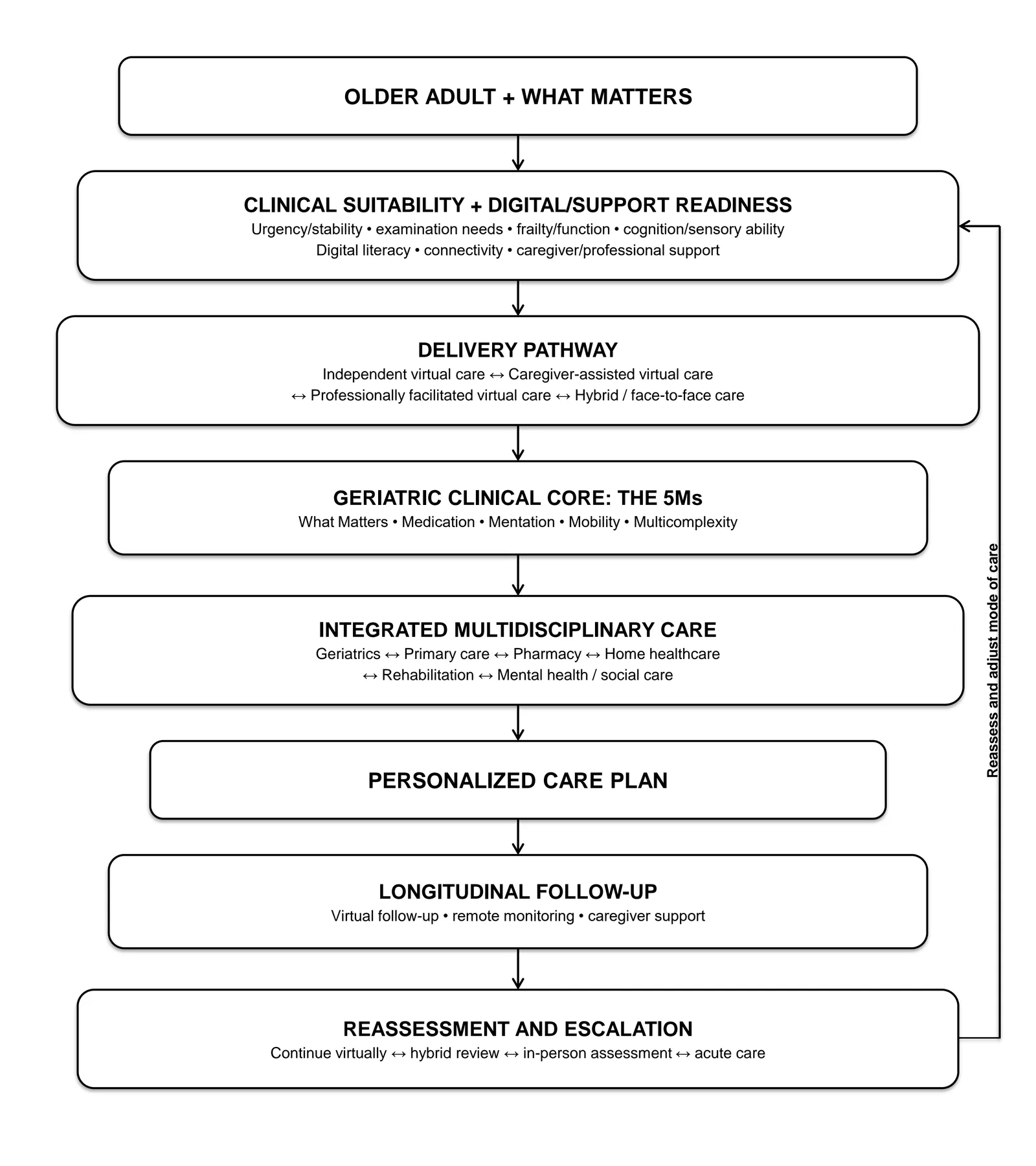
Proposed 5M-based age-friendly telegeriatrics framework for Saudi Arabia The 5Ms form the clinical core, while clinical suitability, digital readiness, and available human support guide selection of the appropriate model of care. The pathway is dynamic and bidirectional, allowing escalation or de-escalation according to changing clinical needs and circumstances. This evidence-informed framework is proposed by the author and requires prospective validation.

Future directions

Future work should evaluate whether integrated telegeriatrics improves clinically meaningful outcomes rather than merely increasing virtual visit counts. Remote monitoring should have defined clinical responsibility, alert thresholds, and escalation pathways; wearables and sensors should complement, not replace, validated geriatric assessment.

Artificial intelligence may support documentation, risk stratification, medication-safety alerts, identification of functional deterioration, and prioritization of patients for review. Algorithms developed in younger or digitally engaged populations may not perform similarly in frail older adults; validation in representative geriatric populations, transparency, clinician oversight, privacy, and avoidance of digital inequity are therefore essential.

Arabic age-friendly interfaces are another priority. Adaptation should extend beyond translation to simplified navigation, readable text, accessible audio, low-complexity authentication, caregiver access with appropriate consent, and alternatives for people unable to use smartphone applications independently.

Integration with home healthcare warrants prospective Saudi evaluation. Local nursing or allied-health professionals could provide selected hands-on assessments while a geriatric specialist participates remotely. Comparative studies should assess feasibility, patient safety, clinical outcomes, caregiver burden, and total costs rather than assume benefit from the delivery model alone.

Research priorities should shift from asking whether older adults can use telehealth toward determining which older adults benefit, from which telegeriatric intervention, under what circumstances, and at what cost. Prospective Saudi studies should examine hospitalization and emergency utilization, function, quality of life, falls, medication-related outcomes, caregiver burden, equity, and cost-effectiveness. Comparative studies of independent virtual, caregiver-assisted, professionally facilitated, hybrid, and conventional models would be particularly valuable. The proposed 5M-based framework itself should be tested prospectively for feasibility, inter-rater usability, patient and caregiver acceptability, safety of triage and escalation decisions, and association with clinical and service outcomes before it is used as a standardized care pathway.

## Conclusions

Telehealth offers an important opportunity to extend geriatric expertise to older adults who face geographical, functional, or logistical barriers to conventional healthcare. Successful telegeriatrics, however, requires more than transferring a traditional outpatient consultation to a video platform. Multimorbidity, polypharmacy, frailty, cognitive and sensory impairment, functional dependency, and caregiver involvement should all be considered when determining the appropriateness and design of virtual care.

The future of telegeriatrics should not be framed as a choice between virtual and face-to-face medicine. A 5M-based, age-friendly hybrid model provides a framework in which What Matters and geriatric complexity define the clinical goals, while digital readiness and available human support guide how care is delivered. Independent virtual care, caregiver-assisted care, professionally facilitated care, and in-person assessment can function as connected options within one pathway rather than competing systems. For Saudi Arabia, integrating expanding virtual-health capabilities with geriatric, primary care, pharmacy, home healthcare, rehabilitation, and caregiver services provides an opportunity to develop and prospectively evaluate a coordinated model of age-friendly telegeriatrics.

## Appendices

Appendix A: Narrative search and evidence-synthesis transparency

Companion to: Age-Friendly Telegeriatrics: Evidence and a 5M-Based Framework for Hybrid Geriatric Care in Saudi Arabia

Design and Sources

This article is a topic-guided narrative review. PubMed/MEDLINE was the primary bibliographic source; Consensus-assisted discovery and backward examination of selected reference lists were supplementary sources. Publications identified through supplementary discovery were checked against underlying peer-reviewed reports.

Search Period and Concepts

The review considered literature published from January 2015 through September 2026, emphasizing literature from 2020 onward. Reported search concepts were as follows: telehealth, telemedicine, virtual care, remote consultation, remote monitoring, mobile health, tele-rehabilitation, older adults, ageing, geriatrics, frailty, dementia, cognition, comprehensive geriatric assessment, polypharmacy, medication review, caregivers, home care, and Saudi Arabia. These are documented topic concepts, NOT verbatim executable Boolean search strings.

Search-Record Limitation

Complete electronic search histories, exact Boolean strings, field tags, filters, search dates, and hit counts were not retained. They cannot be provided or reconstructed as records of the original search. No updated PubMed search was conducted for this revision. Exact replication of the original search is therefore not possible.

Selection Considerations

Literature was considered when relevant to telegeriatrics, telehealth for older adults, geriatric-care principles needed to interpret virtual delivery, caregiver support, digital inclusion, or the Saudi healthcare context. Reviews, comparative studies, professional guidance, and Saudi primary studies were prioritized where pertinent. These considerations describe the narrative scope; they were not prospectively applied eligibility rules in a formal systematic screening workflow. No study flow counts are available.

Quality and Evidence Directness

No formal study-level risk-of-bias instrument or independent duplicate appraisal was performed. Interpretation considered design, population, intervention, reported outcomes, directness, and limitations described in the source publications. Direct telegeriatrics evidence was distinguished from broader older-adult telehealth research and conventional geriatric-care evidence. Feasibility or acceptability did not establish effectiveness; cross-sectional associations did not establish causality.

Narrative Synthesis and 5M Mapping

Findings were organized by clinical domain and Saudi context. The framework was derived interpretively by linking recurring clinical/implementation considerations to What Matters, Medication, Mentation, Mobility, and Multicomplexity and to independent, caregiver-assisted, professionally facilitated, and hybrid delivery. This was not a predefined formal mapping or validation exercise.

Quantitative Synthesis

No new pooled effect estimates, meta-analysis, meta-regression, or statistical tests were performed. Selected effect estimates were reported from published sources, without recalculation. Variation in designs and outcomes limits cross-study comparisons.

Limitations

This narrative process does not guarantee exhaustive identification of studies and is subject to selection and interpretation bias. Missing original search records limit reproducibility. No formal risk-of-bias appraisal was undertaken. The proposed framework remains conceptual and requires prospective testing.
